# Psychosocial factors contributing to value creation in value-based healthcare: a scoping review

**DOI:** 10.3389/fpsyg.2024.1323110

**Published:** 2024-04-09

**Authors:** Leda Marino, Vincenza Capone

**Affiliations:** Department of Humanities, University of Naples Federico II, Naples, Italy

**Keywords:** value-based healthcare, psychosocial perspective, healthcare organizations, organizational perspective, scoping review

## Abstract

**Background:**

Healthcare systems constantly evolve to improve care quality and resource utilization. One way is implementing Value-Based Healthcare (VBHC) an economic approach. This scoping review aims to identify and describe the literature on VBHC, particularly its psychosocial aspects, to uncover research gaps.

**Method:**

The review followed the PRISMA guidelines for Scoping Reviews. We took the following 14 steps: (a) defining the research question; (b) identifying relevant studies; (c) selecting studies; (d) 15 mapping data; (e) collecting, synthesizing and reporting results. A detailed Boolean search was conducted from January 2021 to August 31, 2021, across APA PsycINFO and PubMed databases using keywords such as “Value-Based Healthcare” and “psychosocial perspective.” Initially, three reviewers screened 70 e-records independently, assessing titles, abstracts, and full-text against the inclusion criteria. Discrepancies regarding the evaluation of the articles were resolved through consensus sessions between the reviewers.

**Results:**

The final review included 14 relevant e-records in English from peer-reviewed sources, focusing on quantitative and qualitative research. From the analysis, four areas emerged: (1) Value chains in Healthcare; (2) Styles, activities, and practices of value co-creation in Healthcare; (3) Value co-creation in the encounter process; (4) Value co-creation in preventive health services.

**Conclusion:**

The scoping review findings suggest several potential key aspects, including the interdependence between patients and healthcare organizations, organizational culture in healthcare, and the role of patient-centered approaches that focus on relationships, communication, and social support in healthcare. This can be achieved through patient engagement, patient-centered care and communication, health literacy, psychosocial support services, comprehensive psychosocial assessments, care coordination, and continuity of care. Integrating psychosocial elements in VHBC enhances quality and optimizes resource use. Findings highlight the need to develop practical guidance on how to implement a culture of value in care that takes into account the psychosocial aspects that have emerged, but not fully addressed. The pandemic teaches that the workforce poorly receives sudden and unsystematic changes. This review could provide an initial basis for the redesign of value in healthcare and a paradigm shift that has already begun with patient-centered medicine and patient engagement.

## 1 Introduction

Value-Based Healthcare (VBHC) is an economic approach that prioritizes patient outcomes over service volume to enhance care quality and optimize resource allocation. It originated from the works of Porter and Teisberg (2006), Porter (2009), Porter (2010) and Porter and Lee (2013). The approach focuses on aligning financial incentives with patient results for greater healthcare efficiency. VBHC traditionally focuses on continuously measuring health outcomes and relative costs. It emphasizes the importance of considering the distribution of resources to the population (allocative value), the appropriateness of resource use for specific health needs (technical value), and the alignment of health outcomes with patient expectations (personal value) (Kaplan and Witkowski, 2014). The cost of care for a specific condition is the sum of all related expenses (Depla et al., 2023). According to literature (Beard et al., 2020), hospitals treating chronic diseases such as diabetes can benefit from shifting to VBHC. This can result in better health management, healthier lives for patients, and reduced costs by emphasizing preventive measures and efficient treatments, reducing unnecessary procedures and overall healthcare expenditure. VBHC measures value by assessing the full care cycle, including the patient’s condition, diagnosis, outcomes, satisfaction, and related aspects (Gray, 2018). This requires calculating the costs and effectiveness of resources used. However, evaluating value solely based on costs is insufficient. According to a scoping review by Gunawan et al. (2022), most implementations of VBHC focus only on outcomes and costs, neglecting its multidimensional nature. None of the studies reported complete success in applying all aspects of VBHC. Therefore, hospital leaders need to understand that VBHC adoption involves more than just partial implementation (Gunawan et al., 2022). While traditional discussions of VBHC have emphasized its economic aspects, as noted by Kim et al. (2013) and Kaplan and Porter (2011), recent literature suggests the need to explore its psychosocial dimensions (van Engen et al., 2022).

### 1.1 Healthcare and psychosocial aspects

Healthcare is not only about medical treatments, but also about interpersonal relationships, communication and patient experiences within the healthcare system (Lewis, 2022). Therefore, when considering the value of healthy organizations, it is important to focus on the psychosocial component and the contribution of human resources. The psychosocial aspects of healthcare (Caprara et al., 2019), including patient satisfaction, mental well-being, communication, social support, shared decision making and patient engagement, are crucial in shaping the healthcare experience and have a direct impact on patient outcomes, treatment adherence and healthcare utilization patterns (Beach et al., 2006; Epstein and Street, 2011; Groene, 2011; Capone, 2016; Barello et al., 2021; Gorli and Barello, 2021). The literature (Virlée et al., 2020) highlights the importance of going beyond purely economic measures and considering the relational, psychological and social aspects of care. Such an approach can potentially improve patient outcomes, the well-being of healthcare workers and organizational culture (Capone, 2022; Marino and Capone, 2023). In summary, despite the increasing adoption of VBHC (Gunawan et al., 2022), defining, measuring and understanding the value of healthcare is a challenging task that requires the inclusion of psychosocial dimensions and consideration of the common matrix of social and economic issues (Osei-Frimpong, 2016). This scoping review aims to highlight the psychosocial dimensions of VBHC by describing the potential psychosocial factors that contribute to value creation in healthcare, with a consequent reduction in costs and considerable advantages for the management, also economic, of health and public health organization. In summary if properly systematized and implemented, have the potential to transform healthcare delivery and improve outcomes for all stakeholders (Teisberg et al., 2020).

## 2 Method

Given the exploratory nature of the study, a scoping review seemed the most appropriate choice (Schettino and Capone, 2022). It was conducted according to the Preferred Reporting Items for Systematic Reviews and Meta–Analysis–Scoping Review (PRISMA-ScR) (Tricco et al., 2018) checklist to ensure a systematic and consistent scoping review. After a preliminary search of the scientific literature, we focused primarily on the Value-Based Healthcare traditional framework to extend the working context progressively. We have started with the following steps: (a) defining the research question; (b) identifying relevant studies; (c) selecting studies; (d) charting the data; (e) collecting, summarizing, and reporting the results. Initially, we defined a clear research question, forming the foundation of the entire review. Subsequently, we identified relevant studies through a literature search across the choice databases, employing specific keywords and inclusion/exclusion criteria that involved with the research question. Then, the study selection phase involves screening titles and abstracts for relevance, followed by a full-text review of eligible studies, applying the criteria set. The data was charted by extracting key information from each study, such as author, year, methodology and findings. The final stage encompassed collecting, summarizing, and reporting the results and synthesizing the data to identify themes, gaps, and potential areas for future research. This process is furthered by frameworks such as Arksey and O'Malley (2005), focusing on the breadth of scope; Levac et al. (2010), enhancing suggestions related to the extraction process; and Tricco et al. (2018), addressing advancements in scoping review practices, particularly in database searching. Following the triple-review model, each reviewer evaluated the articles separately to ensure a complete, impartial, and reliable evaluation. Regarding the field of psychosocial health, a scoping review (Augustinavicius et al., 2018, pp. 3–4) recommends involving multiple reviewers in the valuation article’s process for evaluating the relevance of academic articles and to resolve eventual discrepancies.

Regarding the registration on open science framework, we adopt the following criteria: scoping reviews, as defined by Arksey and O'Malley (2005), are a form of knowledge synthesis which addresses broader topics where many different study designs might be applicable. Different from systematic reviews, which have a well-established tradition of prospective registration to enhance transparency and reduce publication bias (Moher et al., 2009), it is possible for scoping reviews not to follow this protocol. This distinction arises because scoping reviews often have more flexible methodologies, which evolve as the review progresses, making prospective registration less practical (Peters et al., 2015). Levac et al. (2010) highlight that scoping reviews are particularly useful for not being extensively reviewed before, which may necessitate adjustments to the review protocol as new insights emerge (Tricco et al., 2018). This is aligned with the lack of a mandatory requirement for methodological quality assessment or risk of bias of included studies typical for the unique objectives of scoping reviews (see Section 2.5). Therefore, while transparency in research is important, the unique characteristics and developmental stages of scoping review methodologies provide a valid rationale for not adhering to the prospective registration process typically associated with systematic reviews (Peters et al., 2021). However, we included this aspect in the limits (see Section 4.1).

### 2.1 Research question

The research methodology employed a bottom-up approach (Gregory, 1990) utilizing the VBHC’s framework. This approach guided the development of specific research questions aimed at exploring the existence of psychosocial dimensions of VBHC. These questions are:

1. Does a psychosocial perspective exist within the VBHC framework?
2. How is this perspective characterized?
3. What key psychosocial factors significantly impact the perceived value in healthcare?

In alignment with the PICO strategy described by Giorgi et al. (2020), the research delineated its scope as follows:

- Target population: included both patients and healthcare professionals.
- Focus of the study: aspects of well-being and factors contributing to discomfort.
- Outcomes: related to the perceived value in healthcare.
- Methodological approach: quantitative and qualitative research designs were employed.

These elements underpin the research’s primary objective: to describe a non-economic, specifically psychosocial, viewpoint within healthcare organizations.

### 2.2 Search strategy

To determine the most suitable search strategy, three specialists in the fields of health and organizations were consulted. Relevant articles were identified through three electronic databases: APA, PsycINFO and PubMed. Thus, the electronic records were identified utilizing a bibliographic search conducted by inserting the algorithm of keywords such as: “Value-Based Healthcare,” “psychosocial perspective”; “psychological variables,” and Boolean operators (“AND,” “OR,” and “NOT”). An example search strategy: Title-Abs-Key (Value-Based Healthcare OR Value-Based Health Care AND psychosocial perspective OR psychological variables) AND [LIMIT-TO (Language, “English”)] (date of last research: 31 August 2023).

### 2.3 Inclusion and exclusion criteria

The peer-reviewed articles were selected on the basis of the following inclusion criteria: (1) qualitative and quantitative empirical studies, (2) written in English, (3) with full-text available online, (4) with clearly defined and explicit methods (5) with clearly implications. Gray literature articles, letters to editor, conference’s abstracts, commentaries and book chapters were excluded, too. Furthermore, articles on topics that are too distant were excluded, for example in-depth field: (e.g., laparoscopic surgery); tool’s specificity (e.g., digital devices); distant theoretical frameworks (e.g., psychiatric focus); purely economic aspects (e.g., drug-related expenses); general services (e.g., tourism) (see Flow Diagram).

Due the exploratory aim of study, no temporal or geographical restriction were used. The origin and the type of health organization were reported in the Table 1. Beyond this, is necessary to specify some aspects of our reflection process that could clarify our choices. Below, we attempt to summarize them.

1. Articles without VBHC Mention: While the primary focus of our research is on Value-Based Healthcare (VBHC), articles that not explicitly mentioned VBHC were still considered if they had clearly defined methods and implications aligned with the broader themes of VBHC to ensure a comprehensive description of the field, including perspectives and methodologies that might indirectly contribute to VBHC discourse.
2. Meaning of “Implications”: By “implications,” we refer to the practical or theoretical consequences of the study’s findings that differ from “results,” which are the direct outcomes of the research. Implications are more about how the results can be interpreted or applied in a broader context, including their relevance to VBHC principles.
3. Specific Outcomes Sought: The study was particularly interested in outcomes related to integrating psychosocial factors in healthcare, patient-centered care, and the effectiveness of healthcare services from a quality and cost perspective. These outcomes were sought to understand how they intersect with VBHC principles.
4. Articles Excluding Psychosocial Factors: If an article did not include explicit psychosocial factors, were it was determine if they it offered significant insights or methodologies that could indirectly contribute to understanding the role of psychosocial factors in VBHC.
5. Identification of Psychosocial Factors: Psychosocial factors were identified through a careful literature review. These factors included but were not limited to patient, healthcare provider attitudes, perceptions, experiences, social support systems, and the impact of these factors on healthcare outcomes.

**Table 1:** Description of included studies.

References	Aim	Participants	Design	Tool	Main results
**Area 1: value chains in healthcare**					
Barnabè and Perna (2019)	To investigate the impact of Lean Thinking activities on the healthcare organization’s performance; determine if these activities can create value for patients and stakeholders while improving cost-effectiveness; examine whether Lean Activities assist healthcare decision-makers in solving managerial problems. Furthermore, seek to enhance the system for measuring organizational performance to evaluate the impact of Lean Activities and the effectiveness of this approach by quantifying the monetary value it enables the organization to recover. Finally, assess the impacts of Lean organizational management and performance over 3 years (2013–2016) regarding the recovered value	1,230 healthcare workers of Hospital of Siena City (AOUS) Department’s	Case study and action research	107 improvement projects through participant observation; in-depth interviews; surveys; performance measurement on the Balanced Scored Card model targeting 4 main dimensions patient; processes; learning and growth; finances, each analyzed through key specific indicators (Key Performance Indicators)	Value creation in terms of quality emerged as the organization’s ability to exploit synergies between operational and socio-technical dynamics, resulting from personnel management through Lean practices, which, in line with Porter, also provided a margin of recovery of financial resources. The proper measurement and evaluation of Lean initiatives were implemented and tested, creating the “value creation capacity” measurement model, which has proved effective and could be applied in other healthcare organizations. Significant results include reducing waiting times for patients (5,383 h recovered) and reducing physical kilometers traveled by streamlining healthcare actions (154 km per process). Regarding the quality of care and patient safety, 107 practice improvement projects have significantly impacted, with standardization of procedures (145 new procedures between checklists, operating instructions, protocols, and agendas). Dysfunctional aspects of processes were reduced (966 units), and 93 projects significantly impacted the learning and growth of professionals and positively impacted organizational well-being
European Society of Radiology (2021)	To detect the importance of assessing how patients perceive the value of radiology services, particularly in the context of Value-Based Radiology (VBR) in Europe. The goal is to align radiology’s value with Value-Based Health Care (VBHC) metrics and optimize radiology services for patients	400 patients from 22 countries, including European Union members, select economic community members, countries with partnership agreements with the EU, and Schengen countries. The list of countries includes Australia, Austria, Belgium, Bulgaria, Canada, France, Germany, England (pre-Brexit), Switzerland, Iraq, Ireland, Italy, Liechtenstein, Nigeria, Netherlands, Portugal, Croatia, Czech Republic, Russia, Spain, Turkey, and the USA	Quantitative Cross-sectional	An anonymous questionnaire was created online using Survey Monkey™ and distributed through various channels, including radiology department heads, European Society of Radiology officials, patient associations, and social media platforms like Facebook. The questionnaire included sections about patient demographics, medical history, satisfaction with care (e.g., radiology service evaluation, staff courtesy, information provided, waiting times), familiarity with value-based radiology, and attitudes toward value (e.g., cost, efficacy, safety, service). Responses were collected on a 5-point Likert scale and through multiple-choice questions. The survey took place from January 29 to June 28, 2019, and was available in multiple languages. Most participants were satisfied with the services. Additionally, there were options for an editable or printable PDF version for paper-based completion, and the study involved participatory observations and in-depth interviews with key stakeholders	The results showed that younger respondents preferred the online mode, with over 50% of them being under 50 years old. In contrast, 25% of paper-based respondents were over 50. Two-thirds of the participants were women. France had the highest response rate with 158 participants. Most respondents had received 1–5 radiological services in the past 2 years, and their overall satisfaction with these services was high, averaging 4.22 out of 5. Almost all participants reported being satisfied or very satisfied with the services they received
Fjeldstad et al. (2020)	Explore, within the Learning Health System an organizational architecture aimed at healthcare based on three interdependent aspects of value creation: value shop, value chain, and value network	Case 1. A kidney transplant patient who became a “trainer” and a group of patients managed by a nurse-educator; Case 2. 160 minor patients and their families	Observational and descriptive analysis of two case studies: Case 1. self-dialysis unit at Ryhov Hospital in the city of Jönköping (Sweden), this service is regionally managed and tax-funded. Case 2. service network for chronic inflammatory bowel disease at the Children Hospital and Medical Center of the City of Cincinnati (State of Ohio in the USA), this service is funded by public and private resources	Participatory observations and in-depth interviews with key stakeholders	Patients who have undergone dialysis and received kidney transplants, when properly trained, can take charge of their treatment and even assist others in self-dialysis units while collaborating with healthcare professionals. This approach enhances healthcare value across three dimensions (individual, organizational, and systemic) by involving patients in all aspects of care, enabling them to manage their health, acquire and share skills, and engage in problem-solving and networking. Healthcare professionals oversee and guide this process, aligning actions with the shared goal of patients’ well-being. Leaders facilitate access to resources and training, ensuring effective management. Benefits include improved individual care, standardized treatment, collaborative networks, and efficient information systems. Healthcare staff observed lower infection rates and higher patient satisfaction. Costs decreased due to better resource allocation among patients, doctors, and researchers. Technological support in the ImproveCareNow network further promoted collaboration and continuous knowledge sharing
**Area 2: styles, activities, and practices of value co-creation in healthcare**					
McColl-Kennedy et al. (2012)	To explore health customers’ engagement in value co-creation practices by identifying the perceived role of the customer, a wide range of customer activities, and the interactions they perform; to explore the relationship between CVCPS (value co-creation practice styles) and quality of life	Director of nursing, 4 oncologists, 2 reception supervisors, and 20 patients from two cancer treatment clinics located in a major Australian city	Qualitative Longitudinal	Depth interviews (comparative method); observational studies in the field; 4 focus groups of 2 h duration: two focus groups with patients undergoing cancer treatment for less than 6 months and two with patients undergoing treatment for more than 6 months. The collection lasted 2 years	Eight macro-themes of value co-creation activities were identified, including cooperation, information gathering, combining complementary therapies, actively seeking and sharing information, making changes in actions, connecting with various individuals and groups, and engaging in co-production and self-improvement activities. The quality of life was assessed in four domains: existential, psychological, supportive, and physical. Five styles of practice in co-creating client health value were identified: group management, controlling/distancing, collaborative, pragmatic/adaptive, and passively compliant. These styles differ in the intensity and type of interactions and exchanges across the four quality of life domains
Ng et al. (2018)	Analyzing how resources and service gaps, in the context of chronic illness, influence care styles in the co-creation of value between caregiver and health client in terms of relationship, health care, and financial planning based on 5 care delivery styles integrating different resources: Delegate; Mentor; Partner; Coach and Validator	402 health clients (57% M) aged 75 years who were also referred to an Australian residential care service for the financial management of their health; 396 chronically ill patients (58.4% F) aged 24–75 years in an Australian context. Over 60% of the sample lived in a married arrangement, and over 45% had a high level of education	Quantitative Cross-sectional	Two online questionnaires were used to explore the relationships between health clients and managers, as well as between patients and doctors, focusing on care and financial aspects. The questionnaires included a socio-diagnostic section and items assessing health clients’ resources, such as physical condition, service-related skills, risk tolerance, awareness, and financial health. Responses were rated on a 7-point Likert scale, except for personal net worth and counseling costs, which required numerical inputs. The severity of health conditions was rated on a scale from 1 to 100. Lack of resources was identified by coding certain items in reverse. Additionally, frequency testing was conducted to understand the distribution of different care provider styles (41 styles) within the sampled populations	The results reveal that among healthcare clients, 26% exhibit a Mentor style, and the same style is observed in 24% of patients. The Partner style is equally prevalent in both financial and healthcare groups, at 25%. The Coach style is more common among financial clients (22%) compared to patients (15%), while the Delegate style is found in 11% of healthcare clients and 7% of patients. The Validator style represents 16% of healthcare clients but is higher at 29% among patients. In terms of resources, Validator clients have a strong sense of belonging (physical resource) and high service-related skills and education (cultural resource). Coach clients have strong personal commitment (physical resource), while Partner clients exhibit the highest risk tolerance (cultural resource). On the other hand, Delegate clients lack a sense of belonging and personal commitment (physical deficiency), and Mentor clients have lower education and risk tolerance (cultural deficiency). Overall, health clients experiencing Validator, Partner, and Coach relationship styles tend to have more resources. In contrast, those with Delegate and Mentor styles have resource deficiencies and rely more on healthcare and management professionals. Specifically, Validator clients perceive their health situation as less complex and have high risk tolerance (cultural resources) but have less severe health conditions (financial resource). Partner clients demonstrate strong personal commitment and service-related skills. In contrast, Delegate clients experience deficiencies in cultural and financial resources, while Mentor clients exhibit higher personal commitment.
Sweeney et al. (2015)	To explore health customer value co-creation activities (EVCA) by identifying a hierarchy of value co-creation activities that require increasing effort (and represent increasingly difficult tasks) and to demonstrate the links between customer EVCA and quality of life, satisfaction with a health care service, and behavioral intentions	20 chronic patients from two cancer clinics for the qualitative interviews and 1,008 patients for the administration of the items (304 with cancer-related diseases; 248 with heart diseases, and 365 from the diabetic area)	Quali-quantitative Cross-sectional	Interviews with open questions lasting about one and a half hours to identify emerging themes related to value co-creation activities. These themes were then analyzed using Rasch analysis with a selection of 12 items, using RUMM 2030 software. Hypotheses were tested using SPSS. Standardized scales in the quantitative survey included a 4-item quality of life measure, a 4-item service satisfaction scale, and a 4-item behavioral intention measure. Responses were rated on a 7-point Likert scale, ranging from 1 (strongly disagree) to 7 (strongly agree)	The qualitative study identified 13 value co-creation activities, such as sharing information, decision-making involvement, interactions with clinical staff, and more. These activities occur within the healthcare organization and through personal and market sources. Then, 12 items corresponding to these themes were selected, and five hypotheses were formulated based on the literature. All hypotheses were confirmed, indicating that value co-creation activities improve the client’s quality of life, service satisfaction, and health-promoting behavior intentions. Satisfaction with the service also partially mediates these relationships
Virlée et al. (2020)	To investigate why some health service users are more able and/or willing to perform resource integration activities than others and what factors at 1.individual, 2. relational, and 3.systemic levels act as facilitators and/or inhibitors of health service users’ resource integration activities and how an inhibiting or facilitating factor affects resource integration activities, and how these abilities relate to users’ well-being	23 respondents, including patients undergoing treatment after a lung transplant and their families; health professionals, and others each representing a case	Multiple case study	Semi-structured interviews were conducted with patients and various stakeholders involved in the healthcare network, including internal and external facilitators. These interviews followed a guide structured around the three phases of the patient pathway: pre-transplant, transplant, and post-transplant phases. Patients shared their experiences during these phases, detailing the activities they undertook and the roles of the five most significant stakeholders in their journey. Subsequently, the stakeholders identified by the patients were interviewed to gather their perspectives on the lung patient pathway, and their input was discussed during service meetings	At the individual level, health literacy positively impacts compliance with care rules, co-production activities, and dietary exercise among health service users. User involvement behaviors also positively influence co-learning with caregivers regarding chronic disease and lifestyle. At the relational level, service provider responsiveness positively affects user compliance with value co-creation and co-learning activities. Additionally, a physician’s ability to provide reassurance and confidence positively influences ongoing care and co-learning/co-creation activities Emotional competence and communication skills of caregivers play crucial roles in influencing the value of co-production activities. Systemically, the physical proximity of users and caregivers for prescription adherence is essential, while collaboration barriers hinder continued treatment. Social support from non-professional stakeholders positively impacts treatment, co-learning, lifestyle changes, diet, exercise, and leisure activities. Overall, well-being is positively influenced by the integration of resources at all levels (individual, relational, systemic), with greater patient and stakeholder commitment leading to higher levels of user well-being
**Area 3: value co-creation in the encounter process**					
Osei-Frimpong (2016)	To examine the influence of the needs of the actors during the consultation meeting and the key elements that produce value and satisfaction in the patient and to analyze the moderating effects of patient characteristics in the encounter process and how these influence the experiential value of the actors	332 outpatients from two clinics in Accra (Ghana)	Quantitative Cross-sectional	A self-report questionnaire comprising several scales and sections, including a Personal Data Sheet with information on the frequency of doctor encounters. The scales used in the questionnaire included the PreEncounter Patients Value Needs Scale, Shared Decision Making Scale, Trust Scale, Care Delivery Approach Scale, Experiential Value Scale, and Satisfaction Scale	The results reveal that patients’ positive expectations about their encounter with the physician have a positive impact on the consultation experience, leading to comfort, reassurance, appropriate prescriptions, and patient-centered care. The doctor’s approach is correlated with the patient’s perceived experiential value, and both factors contribute to patient satisfaction. Patient age, cultural background, and the frequency of doctor encounters act as moderating factors in the physician-patient encounter, particularly affecting trust, shared decision-making, and experiential value. Interestingly, patient involvement in decision-making did not affect perceived experiential value
Osei-Frimpong and Owusu-Frimpong (2017)	To analyze the dyadic interactions between user and care provider to gain an understanding of how these processes and experiences of encounter are perceived by actors to influence the effectiveness of public and professional services	34 outpatients and 10 doctors from three public health facilities in Accra (Ghana)	Qualitative cross-sectional	Semi-structured interviews explored the process of encounters in the doctor’s office, experiences, and how these influence the overall value created. Doctors were recruited first, and then three outpatients were selected. On average, each interview lasted 55 min	Both doctors and patients viewed the nature of their engagement in the consultation as crucial. Doctors believed that accurate diagnoses required patients to speak openly and provide detailed information. However, patients followed this advice only when they had a positive and trustworthy perception of the doctor. Encounters where patients felt rushed and faced a barrage of questions without communication space had negative outcomes. Unclear roles for both doctors and patients were obstacles to participation during consultations Key elements for value co-creation included collaboration, cooperation, interactions, trust, information sharing, and active patient participation. Patients aimed for a better quality of life, which they considered valuable beyond their interactions with the doctor. Meanwhile, doctors prioritized their patients’ recovery as a significant value, as it would enhance patient well-being and reduce healthcare costs
Osei-Frimpong et al. (2015)	To investigate the processes of value co-creation in the doctor-patient dyad and how their experiences in the consultation room influence the value arising from the encounter	8 doctors and 24 patients at a hospital outpatient clinic in Ghana	Qualitative Cross-sectional	45-min semi-structured interviews involving 76 critical incidents Respondents were asked to recall and describe, from both a favorable and unfavorable point of view: situations in which the doctor-patient encounter influenced their experience of the service; elaborate on the event that occurred; how the incident was handled; how the incident affected their experience, perception and value outcome of the service provision	Thematic analysis revealed three key categories influencing the value creation process in healthcare: Social Context: This includes elements like physicians’ social skills (friendliness, empathy, and respect), the level of interactions among healthcare actors, and their communication abilities, which were challenging for physicians. Emotions, trust, and confidence of actors also played a significant role in their experiences Collaboration: This category encompasses actor involvement in decision-making, caregiver-patient orientation, and patient correspondence Value in Healthcare: From both doctors’ and patients’ perspectives, value in healthcare is linked to factors such as recovery, receiving optimal care, involvement in decision-making, positive consultation experiences, understanding the patient, accurate diagnosis, appropriate prescriptions, patient adherence to treatment plans, and overall patient satisfaction. Additional crucial elements include positive experiences, recognizing the patient’s role in care management, reducing complications and consultation visits, and enhancing well-being
Osei-Frimpong (2017)	To examine the influence that different types of motivation have on the participatory behavior of patients in the encounter with the physician for co-creation of value in services and to evaluate how participation influences value outcomes	345 patients of a semi-governmental health facility in Accra (Ghana)	Quantitative Cross-sectional	A self-report questionnaire consisting in a socio-diagnostic form; Intrinsic motivation scale, External regulation scale and Identified regulation scale; Patient participation scale; Commitment to compliance scale; Perceived value realized scale, all assessed on a 5-point Likert scale of agreement	The results indicate that intrinsic autonomy-oriented motivation positively impacts patient participation and commitment to compliance with the physician’s instructions. Extrinsic (externally regulated) motivation also positively influences patient participation but does not significantly affect commitment to compliance. Internal processes mainly drive patients’ commitment to treatment. Active patient participation in doctor encounters does not directly impact commitment to compliance, but both active participation and successful adherence to instructions significantly affect the overall perception of value realized in the doctor-patient encounter
**Area 4: value co-creation in preventive health services**					
Zainuddin et al. (2011)	Investigating the non-economic health value attributed by the consumer to a screening service, adopting a wellness and not a disease perspective	25 women, aged between 50 and 69, belonging to a free breast cancer screening service located in Queensland (Australia). One woman had her first experience of using the service, seven women had been using the service for less than 10 years, and 17 had been using the service for 10 or more years	Qualitative cross-sectional	25 in-depth interviews about the experience of a free breast cancer screening service, processed using an interpretive social marketing (ICR) approach	Six main themes emerged related to Convenience; Control; Peace of Mind; Behavior as Belief Reinforcement; Self-Identification as Having Influence; Benefits for Others. These themes contributes to reducing the burden on the public health system and societal costs associated with treating cancer patients
Zainuddin et al. (2013)	To investigate the role of different actors in the health value creation process and to observe the impact of key service outcomes on health client satisfaction and their intention to adopt preventive behavior	797 Australian women aged 50–69 years attending a breast cancer screening service	Quantitative cross-sectional	The survey employed various items adapted to its context, measured on a 5-point Likert scale (1 = strongly disagree to 5 = strongly agree) about the following areas: Key Service Outcomes; Organizational Inputs; Functional Value; Emotional Value; Health Client Inputs	The following hypotheses were formulated and confirmed: Satisfaction (H1) has a significant and positive relationship with behavioral intentions Functional value (H2) and emotional value (H3) have significant and positive relationships with satisfaction Interpersonal quality (H6) has a significant and positive relationship with emotional value for users Motivational leadership (H8) has a significant and positive relationship with functional value in health services Stress tolerance (H9) has a significant and positive relationship with emotional value However, H4 (Administrative quality) and H7 (Co-production) were not confirmed
Zainuddin et al. (2016)	Exploring self-creation of value and self-management of the health client in a bowel screening service	378 health clients participated in the Australian bowel screening program, 44.6% female and 55.4% male	Quantitative Cross-sectional	Items related the Functional value; Emotional value; Social value; Behavioral contributions Cognitive contributions; Affective contributions; Consumer readiness and Satisfaction	The study made the following assumptions and found support for most of them: Hypothesis 1: Behavioral contributions positively impact functional value, emotional value, and social value in self-value creation Hypothesis 2: Cognitive contributions positively impact functional value in self-value creation Hypothesis 3: Affective contributions positively impact emotional value in self-value creation Hypothesis 4: Consumer willingness positively impacts functional value and emotional value in self-value creation Hypothesis 5: Functional value, emotional value, and social value in self-value creation have a positive relationship with satisfaction Hypothesis 6: Satisfaction has a positive relationship with behavioral intentions in self-value creation However, H3 and H6 did not receive support from the study’s findings

### 2.4 e-records selection process

The initial search identified 70 potential studies for review. After removing 10 duplicates, the titles and abstracts of 60 articles were independently evaluated for relevance by three reviewers specializing in the psychosocial health field. Each article was screened for inclusion by all reviewers. After a consensus session, 35 studies were dismissed as they not align with the set criteria. The remaining 25 studies underwent full-text assessment, resulting in a further 14 being excluded. Consequently, 14 articles were ultimately selected for the scoping review. Throughout the content analysis phase, the reviewers frequently discussed discrepancies or ambiguities in the study selection process (Haan et al., 2021). This deliberation was repeated to establish agreement on the emerging thematic core concepts.

**Figure 1:**
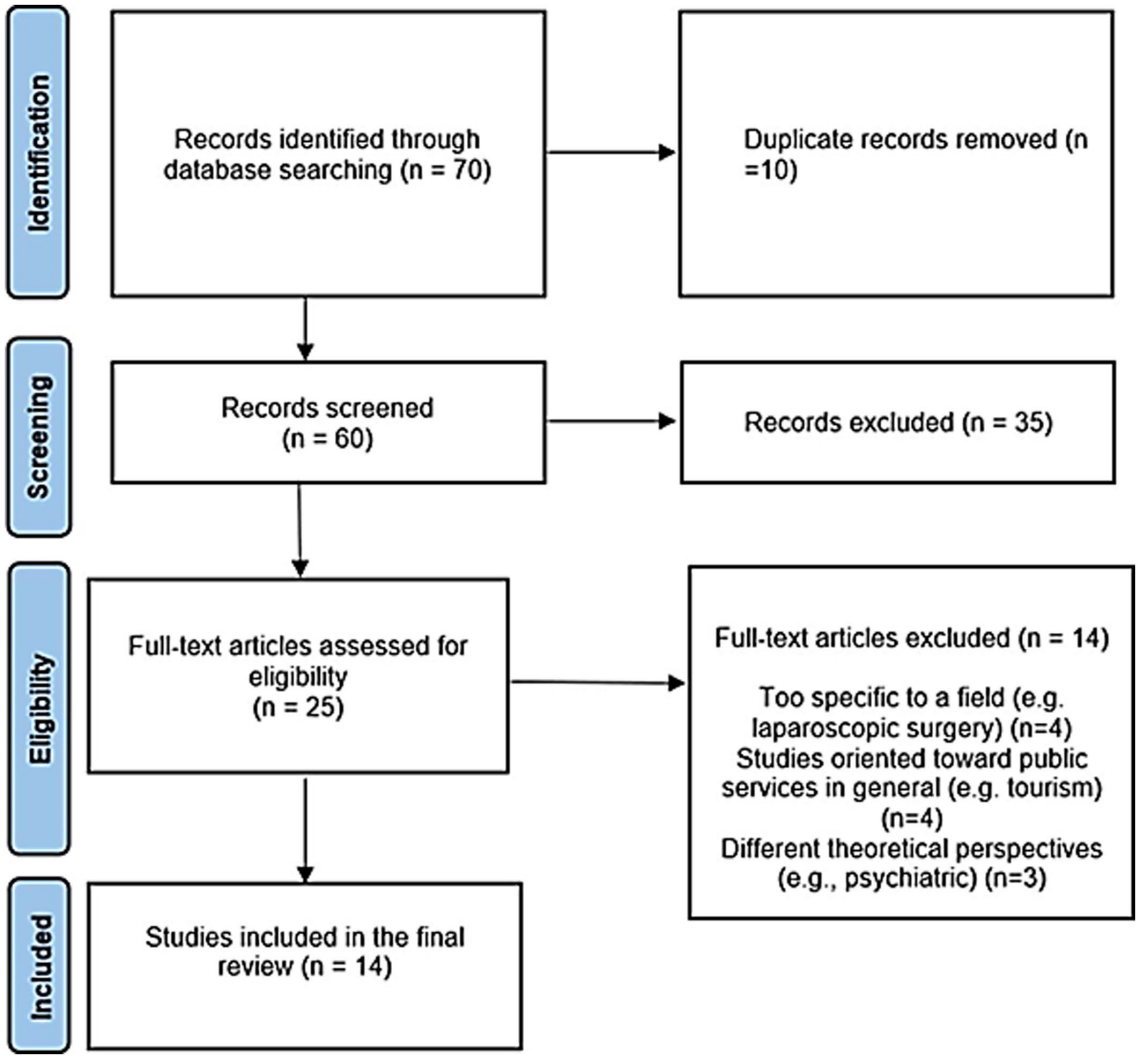
Flow diagram of the literature search strategy and review process, following PRISMA 2020 flow diagram rules.

The flowchart (see Figure 1) illustrates the literature search and screening procedure used in this review. Detailed information on the 14 chosen articles is available in Table 1. The post-analysis categorization of the content of the articles led to the definition of four primary thematic groups, identified by their recurring themes (refer to the Results section).

### 2.5 Quality appraisal

In line with scoping review practice, the included studies have not been assessed in terms of quality (Tricco et al., 2016; Haan et al., 2021). It is worth noting that the primary aim of scoping studies is not to assess the quality of evidence. Indeed, they are recognized for their utility in identifying literature gaps and mapping the breadth of research on a particular topic, as suggested by Arksey and O'Malley (2005). Instead, they focus on exploring the range and nature of research activity in a certain field. This approach is particularly beneficial for topics where the literature is vast or has not been comprehensively reviewed.

## 3 Results

Four main thematic areas were identified:

1. Value chains in healthcare; 2. Styles, activities, and practices of value co-creation in healthcare; 3. Value co-creation in the encounter process; 4. Value co-creation in preventive health services. Table 1 summarizes the studies related to different core concepts that emerged, reporting for each paper, the authors; the description of objectives and participants; the method and tools; and the main results.

### 3.1 Area 1: value chains in healthcare

Studies in this area (Barnabè and Perna, 2019; Fjeldstad et al., 2020; European Society of Radiology, 2021) highlight the essential role of interdependence between the organization and the patient for value creation in healthcare services. In line with the Value-Based Health Care (Porter and Lee, 2013), this determines the effectiveness of outcomes and performance measurement. The need to create value and improve the products and services delivered through care is putting pressure on healthcare organizations and leaders. However, healthcare organizations have been hampered in implementing value processes by the widespread use of cost reduction rather than service redesign (Porter and Teisberg, 2006). Redesigning the healthcare system is critical to achieving effectiveness, efficiency and advances in care, innovation and scientific research. Changing a system’s architecture by increasing investments or reducing costs can lead to sustainable value, but also poses challenges and increases negative outcomes. Examples demonstrate how physicians and patients collaborate as co-producers of healthcare services to create value and achieve positive health outcomes. Personalized care, tailored to individual patient needs, is crucial for optimal results (Epstein and Street, 2011). This can be accomplished using appropriate stakeholders, information, and technologies. For instance, a highly personalized emergency response involving multiple healthcare workers benefits a person with various injuries from a car accident. Recognizing the interdependence between healthcare actors is crucial in pursuing value creation. It is related to the concept of a “value chain” consisting of repeatable and standardized treatment processes used by healthcare workers and patients to achieve desired outcomes which is valuable. Implementing a chain configuration for specific interventions can lead to higher efficiency, improved results, and cost reduction (Barnabè and Perna, 2019; Fjeldstad et al., 2020). However, addressing the issue of differentiation is essential, as standardized solutions may not be suitable for patients with complex medical problems who require personalized care. A value network configuration is proposed to address this challenge, enabling flexible interaction between stakeholders such as patients, physicians, researchers, and organizations. Such networks rely on combinations of platforms and personnel to enhance efficiency and effectiveness (Prahalad and Ramaswamy, 2004). Networked organizations rely less on hierarchy and more on peer collaboration and self-organization, resulting in an “actor-oriented” organizational architecture. This architecture allows for quick adaptation to changing needs through resource reconfiguration. A networked organizational architecture has the potential to facilitate various types of interactions necessary for clinical care, improvement, and research. Shared databases and aggregated knowledge support research accessible to researchers. Integrating of dispersed elements through the network contributes to value chains and the “value shop” concept, which emphasizes personalized responses to patient problems. In the context of the Value Shop, healthcare is based on individual patient-professional relationships. It involves a predictable cycle, including case acquisition, diagnosis development, personalized treatment selection, and solution verification. The value shop prioritizes breadth (managing various medical conditions) and depth (providing expertise) (Gadolin et al., 2020). The increasing complexity of diagnostic and curative interventions, driven by medical knowledge and expectations, has shifted the focus from a single provider of solutions to collaboration among healthcare workers from different disciplines and organizational systems (Rawlinson et al., 2021).

### 3.2 Area 2: styles, activities, and practices of value co-creation in healthcare

Studies in this area (McColl-Kennedy et al., 2012; Sweeney et al., 2015; Ng et al., 2018; Virlée et al., 2020) consider the activities and behaviors of healthcare workers and patients in creating value. In the past, healthcare customers were considered passive recipients outside the realm of the company. However, with the emergence of the Consumer Culture Theory (CCT), customers can now co-create value with the company and its members (Vargo and Lusch, 2016). This shift allows clients to play an active role in the process. Collaborative interactions between individuals and their caregivers are recognized as crucial in effectively managing chronic diseases like cancer, as outlined by the Patient Engagement Model (Graffigna et al., 2020) have the opportunity to integrate resources from healthcare companies, complementary therapies, and private sources such as colleagues, family members, and friends to co-create value (McColl-Kennedy et al., 2012). The co-creation of value is defined as the benefit derived from integrating resources through activities and interactions with collaborators in the client’s service network.

Patients can also co-create value through personal activities and behaviors, such as positive thinking and emotional self-work (McColl-Kennedy et al., 2012). More empirical research is needed on the role of healthcare customers in value creation and its impact on their quality of life, although it is recognized that certain styles of value creation increase organizational productivity. The different approaches to co-creating value are highlighted by McColl-Kennedy et al. (2012). In addition, it has been found that the involvement of users in shared decision-making leads to improved psychological well-being, better medical outcomes and higher levels of satisfaction with the care they receive. The active involvement of users in healthcare management, particularly in the case of chronic conditions, is highlighted by Sweeney et al. (2015). Clients employ use resources beyond healthcare providers, including complementary therapies, private sources, and autonomous positive-thinking activities. For organizational actors, it is important to understand how individuals co-create value to improve their health and well-being.

Sweeney et al. (2015) identified some themes of value co-creation activities, which can be performed within the health facility, through private sources, or market sources. These activities range from information sharing and compliance with basic requirements to positive thinking and emotional regulation. The authors argue that marketing, health psychology, and medical literature support the outcomes for clients and healthcare companies.

Ng et al. (2018) focus on identifying resources that health clients and caregivers can utilize. Resources are knowledge, skills, or personality traits that individuals value for their characteristics or to achieve desired outcomes. Traditional marketing theories primarily consider goods as units of exchange, but recent research recognizes the importance of knowledge and skills in resource integration to create value. Actors involved in a service draw on the resources brought by other actors. Ng et al. (2018) identify several resources relevant to customer value creation in healthcare. These include the sense of belonging, personal commitment, time availability, perception of complexity, service-related skills, risk tolerance, risk awareness, and economic resources. These resources vary among clients and influence healthcare providers’ integration process and activities.

Concerning the last point, Virlée et al. (2020) explain how patients with chronic illnesses co-create value by integrating their resources with various stakeholders. Resource integration activities in healthcare require different skills and efforts from patients and interactions with stakeholders, leading to varying effects on patients’ well-being. In their research, Virlée et al. (2020) explored the factors determining patients’ ability and willingness to engage in resource integration activities and how they relate to their well-being. They identified individual, relational, and systemic factors that act as resource integration facilitators or inhibitors. At the individual level, health literacy and engagement behavior were significant factors. Patients’ knowledge had an impact on their value co-creation activities, specifically their adherence to treatment, co-production activities, and diet and exercise. Previous studies have demonstrated that health literacy impact health behaviors and patient participation in managing their health. In terms of engagement behavior, patients who are more involved in managing their health tend to comply more with involved in medical instructions and engage in autonomous activities. These patients willingly contributed and used resources that affected their well-being.

Several antecedents were identified at the relational level. A crucial role was played by the responsiveness of carers and healthcare teams. This factor had an impact on adherence to prescriptions, mutual learning activities and co-production. Adherence and co-learning activities were also influenced by trust in healthcare workers, especially in relation to assurance. Patients’ adherence to prescriptions and coping activities and their participation in decision-making were influenced by effective communication by health care workers, using understandable language. Factors such as geophysical proximity, system connectivity, and social support were identified at the systemic level. Patients living far from healthcare facilities faced limitations in certain activities, such as physical rehabilitation or regular follow-ups. Lack of system connectivity, including inadequate communication and coordination among stakeholders, posed barriers to patient activities for value co-creation. Social support from non-professional networks, including partners, family, friends, and other patients, significantly influenced patient compliance, co-learning activities, and lifestyle changes. Overall, resource integration activities positively influenced patients’ psycho-physical well-being, as Virlée et al. (2020) highlighted.

### 3.3 Area 3: value co-creation in the encounter process

Studies in this area (Osei-Frimpong et al., 2015; Osei-Frimpong, 2016; Osei-Frimpong, 2017; Osei-Frimpong and Owusu-Frimpong, 2017) highlight how the paradigm shift from passive health clients to patient-centered models bridges a gap in the use of patients’ values, needs, and preferences to guide clinical decisions in service delivery. Research is needed to understand how interdependence in the physician-patient dyad can be a resource for value co-creation (Osei-Frimpong et al., 2015, p. 1).

There are important outcomes of value for healthcare workers and patients. In their study, Osei-Frimpong et al. (2015) state that the actors involved in the encounter have different objectives. Patients perceive value differently based on their expectations. The value of healthcare is conceived as healing, involvement in decision-making, positive experience, understanding the patient, making the correct diagnosis, prescribing appropriate medication, patient compliance, patient satisfaction, and operational efficiency (Osei-Frimpong et al., 2015; Osei-Frimpong and Owusu-Frimpong, 2017). The co-creation of value improves service results, and physicians and patients expressed elements that could enhance the value created during the consultation. These include involvement by the health service, recognition of the patient, reduction of complications or recurrences, and improvement of the health service.

The social context, beliefs and perceptions, and partnership of the dyad are critical areas that support the value co-creation process. The social skills of physicians, level of interactions, and knowledge and skills influence the experiences of physicians and patients. Mutual respect, interpersonal skills, friendliness, empathy, and respect for the patient are important in value co-creation (Osei-Frimpong et al., 2015). The nature of interaction during the consultation process, including listening, explaining, responding, and understanding, influences value co-creation. Two-way communication and active patient participation are preferred over a simple question-and-answer session (Osei-Frimpong et al., 2015). Beliefs and perceptions of patients and healthcare workers, including emotions, trust, and confidence, impact their experiences and value co-creation. Physician reassurance and positive feedback contribute to value creation. Physician-patient collaboration requires active participation and understanding. Patients desire greater involvement in the consultation and a shift away from a paternalistic approach (Osei-Frimpong et al., 2015). Patient adherence to care and compliance with medical instructions positively correlate with value outcomes. Understanding the value needs of the patient before the encounter is essential to reduce conflicts between actors (Osei-Frimpong, 2016). The patient’s commitment to treatment adherence and participation is largely driven by intrinsic motivation. Participation remains central to achieving worthwhile goals, and the social skills of caregivers influence patient participation (Osei-Frimpong, 2017). The patient’s “pre-encounter” expectations and trust influence perceived experiential value and satisfaction. Older patients attribute a higher positive experiential value to shared decision-making. The encounter between physicians and patients is considered a criterion to evaluate the service provision and perceived value. Commitment, information sharing, collaboration, trust, and clarity of roles are essential (Osei-Frimpong and Owusu-Frimpong, 2017). Cognitive and behavioral elements during the encounter produce valuable outcomes. Negative experiences compromise healing, well-being, and positive evaluation of the care service (Osei-Frimpong and Owusu-Frimpong, 2017). Healing, improved well-being, compliance, reduced visits to health facilities, and improved engagement between actors are valuable outcomes of the co-creation process. Elements such as participation, sharing, a relationship beyond prescription, hospital context, and communication contribute to the value created in the physician-patient encounter (Osei-Frimpong and Owusu-Frimpong, 2017).

### 3.4 Area 4: value co-creation in preventive health services

A final area highlighted how even preventive organizational contexts are characterized by actions and behaviors considered “value” by healthcare workers and patients. Studies in this area (Zainuddin et al., 2011, 2013, 2016) emphasize the active role of patients in healthcare, highlighting that preventive health services by society not only diminish public health system expenses but also yield benefits beyond mere cost savings. These advantages embrace emotional well-being and social health, which contribute to the broader functional value of healthcare initiatives. In this sense, healthcare organizations’ demand and supply studies must include “non-economic benefits” by adopting a well-being and pro-activity perspective. Adopting a marketing perspective that is not only aimed at financial gains and competitiveness can be detected both organizationally and for employees and users (Zainuddin et al., 2016). The first study (2011) investigates the value of healthcare from consumers’ point of view, following the social marketing approach in free public prevention services. Six themes emerged from the interviews conducted with the participants, inspired by Holbrook’s classification (Holbrook, 2006):

1. Convenience and health behavior: Convenience and ease of access to health services and facilities were considered fundamental. Practical and structural aspects that facilitate valuable outcomes fall under the functional dimension of value.
2. Control: Participants felt that regular screening and following healthy habits recommended by the prevention service gave them a sense of control over their health. This control is part of the functional and emotional dimensions of value.
3. Peace of mind: Following preventive behavior reduces negative emotions and provides service users with positive emotional reinforcement and reassurance. This aspect contributes to the emotional dimension of value.
4. Behaviors as reinforcement of beliefs: Preventive behavior reinforces individuals’ belief that they act healthily and are healthy. This aspect also falls under the emotional dimension of value.
5. Identifying oneself as having influence: Successfully experiencing preventive health services and adopting healthy behaviors contributes to the social dimension of value. Participants reported encouraging and persuading others to undergo breast cancer screening, creating a virtuous circle.
6. Benefits of one’s behavior for others: Adopting preventive health behaviors impacts micro-contexts and the wider community. It addresses the altruistic dimension of value, reducing costs to the health system and benefiting others.

The study found that the healthcare client’s engagement in value creation increases when the expert figure is less present. This finding strongly influences value creation in services that empower patients. Additionally, the study found that cognitive contributions positively influence emotional value, as well as functional value. Functional value enables healthcare clients to take control of their health by providing practical means to achieve emotional value, which is the desired goal of well-being. In addition, functional value is a stronger key to health client satisfaction than other dimensions, such as emotional value (Zainuddin et al., 2013). Furthermore, social value showed its influence on value creation, especially when health clients with prior experience in self-management of prevention transferred their knowledge about new adherents to preventive protocols (Zainuddin et al., 2016).

## 4 Discussion

This review examines the relational and psychosocial factors in Value-Based Healthcare (VBHC). It is evident that certain psychosocial aspects have emerged but require further development or systematic understanding. The discussion outlines the key differences that underpin our reflections: (1) The interdependence between patients and healthcare organizations should be considered; (2) The organizational culture plays a significant role in shaping healthcare worker interactions and patient care approach; (3) Healthcare should be framed as a client/customer relationship, with a focus on shared decision-making, patient satisfaction, partnership, patient-centered communication, and trust as psychosocial aspects for improving psychological well-being and quality of healthcare while reducing costs; (4) There is a need for a systematic study of non-financial perspectives in healthcare and for systematically assessing efficacy beliefs in health value creation. The review examines the structure of healthcare systems and the interactions between patients, physicians, and researchers. Organizational culture, as described by Schein (1983), is central to this discussion as it influences the dynamics among healthcare professionals, their approach to patient care, and their responses to challenges. These factors collectively shape patient experiences and health outcomes (Barry and Edgman-Levitan, 2012). Furthermore, this text highlights the difference between personalized medicine and patient-centered care. Personalized medicine involves customizing treatments based on individual patient characteristics, whereas patient-centered care emphasizes patient engagement, decision-making, communication, respect, and trust (Hood and Friend, 2011). The latter aligns more closely with a VBHC’s psychosocial perspective.

### 4.1 VBHC’s psychosocial perspective

The following are some of the more specific considerations in relation to the areas that have emerged from the review.

The first area underscores the role of patient-healthcare provider interdependence in shaping effective healthcare delivery and outcomes. The primary aspect we note is the interdependence between patients and health organizations, emphasizing mutual reliance and collaboration for better healthcare outcomes (Porter and Teisberg, 2006). From a psychosocial standpoint, this interdependence fosters patient engagement, increases health literacy, and empowers patients in their healthcare decisions (Hibbard and Greene, 2013). Such an approach is not only in line with the psychosocial framework of VBHC but also aims to elevate patient satisfaction and health outcomes.

The second area of study highlighted the use of terms like “health client” or “customer,” framing the healthcare perspective. It suggests that shared decision-making could improve psychological well-being and satisfaction with care. However, a more systematic investigation needs to examine the positive aspects of mental and psychosocial well-being. Nevertheless, no related evidence regarding mental and psychosocial well-being (Keyes, 2005) has been systematically investigated in VBHC. Moreover, aspects related to positive mental well-being, such as a sense of belonging, health literacy, and physician and patient skills (Capone et al., 2022), are posited but not investigated from a psychosocial health perspective. The studies included in the review also touch on the value co-creation concept and the importance of effective communication in healthcare, underlining the necessity for the perception of efficacy in health communication, as Bandura (1993) and others (Ong et al., 1995; Ha and Longnecker, 2010; Street and Haidet, 2011) have suggested. The review also addresses the role of social support, differentiating between non-professional network support and health organizational support (Uchino, 2006). While the former includes assistance from personal connections like family and friends, the latter involves structured support from healthcare organizations and workers. This support is more formal and structured, focused on meeting healthcare needs, ensuring patient safety, and fostering a supportive work environment for healthcare workers (Capone et al., 2022). Both types are essential, but in the context of VBHC, health organizational support is more significant.

The third area of study shifts to patient-physician relationships, highlighting the importance of aspects such as partnership, trust, and clear communication. These elements are crucial for a patient-centered approach (Epstein and Street, 2011) and align with the concept of patient-centered communication (PCC; Stewart, 1995; Capone, 2016), which is vital for reducing healthcare costs and enhancing the quality of the physician-patient relationship. This approach contrasts with physician-centered communication and improves the quality of the physician-patient relationship by providing clear information, showing empathy, and being expressive in non-verbal language. PCC promotes patient engagement and reduced physician-patient conflict, decreased patient avoidance, and increased satisfaction with the quality of care. Moreover, promoting patient-centered communication can reduce healthcare costs (Hong and Oh, 2020), in line with the economic goals of VBHC.

Finally, the fourth area of study discusses aspects such as “non-economic benefits” in healthcare, particularly preventive health services. This aligns with Bandura’s socio-cognitive theory (Bandura, 2004), emphasizing the role of self-efficacy in health-related value creation. However, there is a need for more systematic studies to assess the role of efficacy beliefs in this process. As mentioned above, efficacy beliefs related to health have a fundamental role in the psychosocial perspective (Bandura, 2018). We acknowledge these studies’ efforts to include a socio-cognitive perspective. However, we must highlight the need to asses studies and tools that systematize the role of efficacy beliefs in the health value creation process from a non-financial perspective because the gap remains. Advancing our understanding of efficacy beliefs in non-economic healthcare can enrich psychosocial discourse and contribute to holistic interventions.

### 4.2 Limitations of the study

As scoping studies do not seek to assess evidence quality, they cannot determine whether particular studies provide robust or generalizable findings (Arksey and O'Malley, 2005). As part of the scoping review process, the articles included in the review were not assessed for accuracy. First of all, we have already discussed in the Methods section (See paragraph 2) the appropriateness of not following the open science registration framework for this scoping review. We also only considered articles in English. This practice can impose limitations on the generalizability of their results: the exclusion of significant research findings published in other languages the overlooking of culturally specific or geographic specific perspectives potentially marginalizing findings from non-English speaking regions, an overrepresentation of viewpoints from English-speaking countries and a lack relevance of different cultural contexts (Meneghini and Packer, 2007; Amano et al., 2016). Furthermore, it is important to note that, as noted by Paez (2017), the removal of gray literature may have introduced study bias. In addition, the use of keywords did not always ensure consistency with subject areas and may have suffered from a subjective criterion that should be better controlled in subsequent studies. Due to the prevalence of the cross-sectional method and the different geographical areas, the results cannot be considered generalizable. Finally, the methodological framework of the work could have been strengthened by including statistical indices of agreement on the thematic categories identified. Nevertheless, this analysis is valuable for exploring peer-reviewed articles in the context of healthcare value.

## 5 Conclusion

The findings of this scoping review will contribute to a better understanding of the psychosocial dimensions of Value-Based Healthcare, inform policy and practice, and identify gaps in the literature for future research. Several studies have indicated, albeit weakly, some psychosocial aspects of value in health that should be further explored and implemented, as shown in this scoping review. In line with the broadening horizons that the literature on value is embracing (Teisberg et al., 2020; Lewis, 2022), we propose a possible future agenda in this regard:

- **Patient Engagement** (Barello et al., 2022): Engaging patients in managing their psychosocial well-being could be a key aspect of VBHC. This may involve providing resources, tools, and interventions to promote self-care, and coping strategies.
- **Patient-Centered Care and Communication** (Sheeran et al., 2023): VBHC could emphasize patient-centeredness, which includes considering patients’ psychosocial needs and preferences. It involves actively listening to patients, understanding their values, beliefs, and goals, and incorporating these factors into the care plan. Effective communication ensures that patients are actively involved in decision-making, leading to improved treatment adherence and better health outcomes. It also promotes patient satisfaction and engagement, which are key indicators of value in healthcare.
- **Health literacy** (Barello et al., 2022): Health literacy empowers individuals to make informed decisions about their health, leading to improved health outcomes. It acts as a bridge between healthcare workers and patients, facilitating effective communication, promoting trust, and fostering a patient-centered care approach. Investing in health literacy initiatives, such as improving health education, should promote clear and accessible health information, enhance communication skills, and prioritize patient well-being and cost efficiency.
- **Psychosocial Support Services** (Capone et al., 2022): VBHC recognizes the importance of providing psychosocial support services as part of comprehensive care. This may include access to mental health professionals, social workers, counselors, or support groups to address patients’ emotional and social needs.
- **Psychosocial Assessments** (Marino and Capone, 2023): Comprehensive assessments of patients’ psychosocial well-being could be integrated into the care process. This may involve evaluating traditional factors such as socioeconomic status, cultural background, and implementing a tool to measure psychosocial dimensions of value in health related to patients and physicians.
- **Patient-Reported Outcome Measures (PROMs)** (Depla et al., 2023): PROMs are tools used in VBHC to assess patients’ perspectives on their health status and quality of life. These measures capture psychosocial aspects, such as emotional well-being, social functioning, and the impact of the illness on daily life.
- **Care Coordination and Integration** (Lewis, 2022): VBHC emphasizes care coordination and integration across healthcare settings, including mental health services, social services, and community resources. This ensures that patients’ psychosocial needs are addressed holistically and that they receive appropriate support beyond clinical interventions.
- **Continuity of Care** (Porter and Kramer, 2019): VBHC recognizes the importance of continuity in the patient-professional relationship. Consistency in healthcare relationships promotes trust, communication, and understanding of patients’ psychosocial needs over time.

## Author contributions

LM: Conceptualization, Data curation, Investigation, Methodology, Project administration, Resources, Writing – original draft, Writing – review & editing. VC: Conceptualization, Investigation, Project administration, Supervision, Writing – review & editing.

## References

[ref1] Amano T. González-Varo J. P. Sutherland W. J. (2016). Languages are still a major barrier to global science. PLoS Biol. 14:e2000933. doi: 10.1371/journal.pbio.2000933 28033326 PMC5199034

[ref2] Arksey H. O'Malley L. (2005). Scoping studies: towards a methodological framework. Int. J. Soc. Res. Methodol. 8, 19–32. doi: 10.1080/1364557032000119616

[ref3] Augustinavicius J. L. Greene M. C. Lakin D. P. Tol W. A. (2018). Monitoring and evaluation of mental health and psychosocial support programs in humanitarian settings: a scoping review of terminology and focus. Confl. Heal. 12:9. doi: 10.1186/s13031-018-0146-0, PMID: 29560023 PMC5858133

[ref4] Bandura A. (1993). Perceived self-efficacy in cognitive development and functioning. Educ. Psychol. 28, 117–148. doi: 10.1207/s15326985ep2802_3

[ref5] Bandura A. (2004). Health promotion by social cognitive means. Health Educ. Behav. 31, 143–164. doi: 10.1177/1090198104263660 15090118

[ref6] Bandura A. (2018). Toward a psychology of human agency: pathways and reflections. Psychol. Sci. 13, 130–136. doi: 10.1177/1745691617699280 29592657

[ref7] Barello S. Caruso R. Palamenghi L. Nania T. Dellafiore F. Bonetti L.. (2021). Factors associated with emotional exhaustion in healthcare professionals involved in the COVID-19 pandemic: an application of the job demands-resources model. Int. Arch. Occup. Environ. Health 94, 1751–1761. doi: 10.1007/s00420-021-01669-z, PMID: 33660030 PMC7928172

[ref9001] Barello S. Paleologo M. Palamenghi L. Acampora M. Graffigna G. (2022). Public perceptions of harms and benefit of covid-19 immunity certificate: a cross-sectional study in the italian setting. Vaccines. 10, 1501. doi: 10.3390/vaccines10091501 36146580 PMC9505085

[ref8] Barnabè G. Perna D. (2019). Assessing performance and value-creation capabilities in lean healthcare: insights from a case study. Public Money Manag. 39, 503–511. doi: 10.1080/09540962.2019.1598197

[ref9] Barry M. J. Edgman-Levitan S. (2012). Shared decision making — the pinnacle of patient-centered care. New Eng. J. Med. 366, 780–781. doi: 10.1056/NEJMp1109283, PMID: 22375967

[ref10] Beach M. C. Inui T. Relationship-Centered Care Research Network (2006). Relationship-centered care: a constructive reframing. J. Gen. Inter. Med. 21, S3–S8. doi: 10.1111/j.1525-1497.2006.00302.x PMC1484841 16405707

[ref11] Beard J. A. Franco D. L. Click B. H. (2020). The burden of cost in inflammatory bowel disease: a medical economic perspective and the future of value-based care. Curr. Gastroenterol. Rep. 22:6. doi: 10.1007/s11894-020-0744-z 32002671

[ref12] Capone V. (2016). Patient communication self-efficacy, self-reported illness symptoms, physician communication style and mental health and illness in hospital outpatients. J. Health Psychol. 21, 1271–1282. doi: 10.1177/1359105314551622, PMID: 25274717

[ref13] Capone V. (2022). Medical communication perceived self-efficacy (ME-CO) scale: construction and validation of a new measuring instrument from a socio-cognitive perspective. Eur. J. Investig. Health Psychol. Educ. 12, 765–780. doi: 10.3390/ejihpe12070056, PMID: 35877456 PMC9323938

[ref14] Capone V. Borrelli R. Marino L. Schettino G. (2022). Mental well-being and job satisfaction of hospital physicians during COVID-19: relationships with efficacy beliefs, organizational support, and organizational non-technical skills. Int. J. Environ. Res. Public Health 19:3734. doi: 10.3390/ijerph19063734, PMID: 35329420 PMC8948767

[ref15] Caprara G. V. Alessandri G. Caprara M. (2019). Associations of positive orientation with health and psychosocial adaptation: a review of findings and perspectives. Asian J. Soc. Psychol. 22, 126–132. doi: 10.1111/ajsp.12325

[ref16] Depla A. L. Pluut B. Lamain-de Ruiter M. Kersten A. W. Evers I. M. Franx A.. (2023). PROMs and PREMs in routine perinatal care: mixed methods evaluation of their implementation into integrated obstetric care networks. J. Patient Rep. Outcomes 7:26. doi: 10.1186/s41687-023-00568-w, PMID: 36894797 PMC9998006

[ref17] Epstein R. M. Street R. L. (2011). The values and value of patient-centered care. Ann. Fam. Med. 9, 100–103. doi: 10.1370/afm.1239, PMID: 21403134 PMC3056855

[ref18] European Society of Radiology (2021). Patient survey of value in relation to radiology: results from a survey of the European Society of Radiology (ESR) value-based radiology subcommittee. Insights Imaging 12:6. doi: 10.1186/s13244-020-00943-x, PMID: 33411144 PMC7790952

[ref19] Fjeldstad Ø. D. Johnson J. K. Margolis P. A. Seid M. Höglund P. Batalden P. B. (2020). Networked health care: rethinking value creation in learning health care systems. Learn Health Syst. 4:e10212. doi: 10.1002/lrh2.10212, PMID: 32313837 PMC7156860

[ref20] Gadolin C. Andersson T. Eriksson E. Hellström A. (2020). Providing healthcare through “value shops”: impact on professional fulfilment for physicians and nurses. J. Health Gover. 25, 127–136. doi: 10.1108/IJHG-12-2019-0081

[ref9002] Giorgi G. Lecca L. I. Leon-Perez J. M. Pignata S. Topa G. Mucci N. (2020). Emerging issues in occupational disease: mental health in the aging working population and cognitive impairment—a narrative review. BioMed Research International. 6. doi: 10.1155/2020/1742123 PMC7011340 32083124

[ref9003] Graffigna G. Barello S. Riva G. Corbo M. Damiani G. Iannone P. Ricciardi W. (2020). Italian consensus statement on patient engagement in chronic care: process and outcomes. Int. J. Environ. Res. Public Health. 17:4167. doi: 10.3390/ijerph17114167 32545278 PMC7312656

[ref21] Gorli M. Barello S. (2021). Patient centredness, values, equity and sustainability: professional, organizational and institutional implications. Sustain. For. 13:23. doi: 10.3390/su132313217

[ref22] Gray M. (2018). Value-based healthcare. Brit. Med. 356:j437. doi: 10.1136/bmj.j437 28130219

[ref23] Gregory R. L. Eye and brain: The psychology of seeing. Princeton: Princetown University Press (1990).

[ref24] Groene O. (2011). Patient centredness and quality improvement efforts in hospitals: rationale, measurement, implementation. Int. J. Qual. Health Care 23, 531–537. doi: 10.1093/intqhc/mzr058, PMID: 21862449

[ref25] Gunawan E. Nadjib M. Soraya S. (2022). Key success in the transformation from volume-based to value-based healthcare: a scoping review. J. Soc. Sci. 3, 986–1002. doi: 10.46799/jss.v3i5.414

[ref26] Ha J. F. Longnecker N. (2010). Doctor-patient communication: a review. Ochsner J. 10, 38–43. PMID: 21603354 PMC3096184

[ref27] Haan R. Alblooshi M. E. Syed D. H. Dougman K. K. Al Tunaiji H. Campos L. A.. (2021). Health and well-being of athletes during the coronavirus pandemic: a scoping review. Front. Public Health 9:641392. doi: 10.3389/fpubh.2021.641392, PMID: 33937171 PMC8085390

[ref28] Hibbard J. H. Greene J. (2013). What the evidence shows about patient activation: better health outcomes and care experiences; fewer data on costs. Health Aff. 32, 207–214. doi: 10.1377/hlthaff.2012.1061 23381511

[ref29] Holbrook M. B. (2006). Consumption experience, customer value, and subjective personal introspection: an illustrative photographic essay. J. Bus. Res. 59, 714–725. doi: 10.1016/j.jbusres.2006.01.008

[ref30] Hong H. Oh H. J. (2020). The effects of patient-centered communication: exploring the mediating role of Trust in Healthcare Providers. Health Commun. 35, 502–511. doi: 10.1080/10410236.2019.157042 30706741

[ref31] Hood L. Friend S. H. (2011). Predictive, personalized, preventive, participatory (P 4) cancer medicine. Nat. Rev. Oncol. 8, 184–187. doi: 10.1038/nrclinonc.2010.227, PMID: 21364692

[ref32] Kaplan R. S. Porter M. E. (2011). How to solve the cost crisis in health care. Harv. Bus. Rev. 89:914. 21939127

[ref33] Kaplan R. S. Witkowski M. (2014). Better accounting transforms health care delivery. Account. Horiz. 28, 365–383. doi: 10.2308/acch-50658

[ref34] Keyes C. L. M. (2005). Mental illness and/or mental health? Investigating axioms of the complete state model of health. J. Consult. Clin. Psychol. 73, 539–548. doi: 10.1037/0022-006X.73.3.539 15982151

[ref35] Kim J. Y. Farmer P. Porter M. E. (2013). Redefining global health-care delivery. Lancet 382, 1060–1069. doi: 10.1016/S0140-6736(13)61047-8 23697823

[ref36] Levac D. Colquhoun H. O'Brien K. K. (2010). Scoping studies: advancing the methodology. Implement. Sci. 5:69. doi: 10.1186/1748-5908-5-69, PMID: 20854677 PMC2954944

[ref37] Lewis S. (2022). Value-based healthcare: is it the way forward? Fut. Health 9, 211–215. doi: 10.7861/fhj.2022-0099, PMID: 36561818 PMC9761467

[ref38] Marino L. Capone V. (2023). Value of care: an exploratory qualitative study with doctors and patients. Eur. J. Investig. Health Psychol. Educ. 13, 1117–1129. doi: 10.3390/ejihpe13070084, PMID: 37504475 PMC10378535

[ref39] McColl-Kennedy J. R. Vargo S. L. Dagger T. S. Sweeney J. C. van Kasteren Y. (2012). Health care customer value cocreation practice styles. J. Serv. Res. 15, 370–389. doi: 10.1177/1094670512442806

[ref40] Meneghini R. Packer A. L. (2007). Is there science beyond English? Initiatives to increase the quality and visibility of non-English publications might help to break down language barriers in scientific communication. EMBO Rep. 8, 112–116. doi: 10.1038/sj.embor.7400906, PMID: 17268499 PMC1796769

[ref41] Moher D. Liberati A. Tetzlaff J. Altman D. G. (2009). Preferred reporting items for systematic reviews and meta-analyses: the PRISMA statement. BMJ 339:b2535. doi: 10.1136/bmj.b2535, PMID: 19622551 PMC2714657

[ref42] Ng S. C. Sweeney J. C. Plewa C. (2018). Managing customer resource endowments and deficiencies for value Cocreation: complex relational services. J. Serv. Res. 22, 156–172. doi: 10.1177/1094670518812195

[ref43] Ong L. M. de Haes J. C. Hoos A. M. Lammes F. B. (1995). Doctor-patient communication: a review of the literature. Soc. Sci. Med. 40, 903–918. doi: 10.1016/0277-9536(94)00155-M 7792630

[ref44] Osei-Frimpong K. (2016). Examining the effects of patient characteristics and prior value needs on the patient-doctor encounter process in healthcare service delivery. Int. J. Pharm. Healthc. Mark. 10, 192–213. doi: 10.1108/IJPHM-01-2016-0005

[ref45] Osei-Frimpong K. (2017). Patient participatory behaviors in healthcare service delivery: self-determination theory (SDT) perspective. J. Serv. Theory Pract. 27, 453–474. doi: 10.1108/JSTP-02-2016-0038

[ref46] Osei-Frimpong K. Owusu-Frimpong N. (2017). Value co-creation in health care: a phenomenological examination of the doctor-patient encounter. J. Nonprofit Public Sect. Market. 29, 365–384. doi: 10.1080/10495142.2017.1326356

[ref47] Osei-Frimpong K. Wilson A. Owusu-Frimpong N. (2015). Service experiences and dyadic value co-creation in healthcare service delivery: an ICT approach. J. Serv. Theory Pract. 120, 82–93. doi: 10.1016/j.jbusres.2020.07.037

[ref9004] Paez A. (2017). Gray literature: an important resource in systematic reviews. J. Evid. Based Med. 10, 233–240. doi: 10.1111/jebm.12266, PMID: 28857505

[ref48] Peters M. D. Godfrey C. M. Khalil H. McInerney P. Parker D. Soares C. B. (2015). Guidance for conducting systematic scoping reviews. Int. J. Evid. Based Healthc. 13, 141–146. doi: 10.1097/XEB.0000000000000050 26134548

[ref49] Peters M. D. J. Marnie C. Colquhoun H. Garritty C. M. Hempel S. Horsley T.. (2021). Scoping reviews: reinforcing and advancing the methodology and application. Syst. Rev. 10:263. doi: 10.1186/s13643-021-01821-3, PMID: 34625095 PMC8499488

[ref50] Porter M. E. (2009). A strategy for health care reform — toward a value-based system. N. Engl. J. Med. 361, 109–112. doi: 10.1056/NEJMp0904131, PMID: 19494209

[ref51] Porter M. E. (2010). What is value in health care? N. Engl. J. Med. 363:26. doi: 10.1056/NEJMp1011024 21142528

[ref52] Porter M. E. Kramer M. R. (2019). “Creating shared value” in Managing sustainable business. eds. Lenssen G. Smith N. (Dordrecht: Springer), 323–346.

[ref53] Porter M. E. Lee T. H. (2013). The strategy that will fix health care. Harv. Bus. Rev. 91, 50–70.23898735

[ref54] Porter M. E. Teisberg E. O. (2006). Redefining health care: creating value-based competition on results Boston, MA: Harvard Business Review.

[ref55] Prahalad C. K. Ramaswamy V. (2004). Co-creation experiences: the next practice in value creation. J. Int. Mark. 18, 5–14. doi: 10.1002/dir.20015

[ref56] Rawlinson C. Carron T. Cohidon C. Arditi C. Hong Q. N. Pluye P.. (2021). An overview of reviews on Interprofessional collaboration in primary care: barriers and facilitators. Int. J. Integr. Care 21:32. doi: 10.5334/ijic.5589, PMID: 34220396 PMC8231480

[ref57] Schein E. H. (1983). The role of the founder in creating organizational culture. Organ. Dyn. 12, 13–28. doi: 10.1016/0090-2616(83)90023-2

[ref58] Schettino G. Capone V. (2022). Learning design strategies in MOOCs for physicians’ training: a scoping review. Int. J. Environ. Res. Public Health 19:14247. doi: 10.3390/ijerph192114247, PMID: 36361125 PMC9657716

[ref59] Sheeran N. Jones L. Pines R. Jin B. Pamoso A. Eigeland J.. (2023). How culture influences patient preferences for patient-centered care with their doctors. J. Commun. Healthc. 16, 186–196. doi: 10.1080/17538068.2022.2095098, PMID: 37401877

[ref60] Stewart M. A. (1995). Effective physician-patient communication and health outcomes: a review. CMAJ 152, 1423–1433. 7728691 PMC1337906

[ref61] Street R. L. Jr. Haidet P. (2011). How well do doctors know their patients? Factors affecting physician understanding of Patients' health beliefs. J. Gen. Intern. Med. 26, 21–27. doi: 10.1007/s11606-010-1453-3, PMID: 20652759 PMC3024116

[ref62] Sweeney J. C. Danaher T. S. McColl-Kennedy J. R. (2015). Customer effort in value cocreation activities: improving quality of life and behavioral intentions of health care customers. J. Serv. Res. 18, 318–335. doi: 10.1177/1094670515572128

[ref63] Teisberg E. Wallace S. O'Hara S. (2020). Defining and implementing value-based health care: a strategic framework. Acad. Med. 95, 682–685. doi: 10.1097/ACM.0000000000003122, PMID: 31833857 PMC7185050

[ref64] Tricco A. C. Lillie E. Zarin W. O’Brien K. Colquhoun H. Kastner M.. (2016). A scoping review on the conduct and reporting of scoping reviews. BMC Med. Res. Methodol. 16:15. doi: 10.1186/s12874-016-0116-4 26857112 PMC4746911

[ref65] Tricco A. C. Lillie E. Zarin W. O'Brien K. K. Colquhoun H. Levac D.. (2018). PRISMA extension for scoping reviews (PRISMA-ScR): checklist and explanation. Ann. Intern. Med. 169, 467–473. doi: 10.7326/M18-0850, PMID: 30178033

[ref66] Uchino B. N. (2006). Social support and health: a review of physiological processes potentially underlying links to disease outcomes. J. Behav. Med. 29, 377–387. doi: 10.1007/s10865-006-9056-5, PMID: 16758315

[ref67] van Engen V. Bonfrer I. Ahaus K. Buljac-Samardzic M. (2022). Value-based healthcare from the perspective of the healthcare professional: a systematic literature review. Front. Public Health 9:800702. doi: 10.3389/fpubh.2021.800702, PMID: 35096748 PMC8792751

[ref69] Vargo S. L. Lusch F. R. (2016). Institutions and axioms: an extension and update of service-dominant logic. JAMS 44, 5–23. doi: 10.1007/s11747-015-0456-3

[ref70] Virlée J. B. Hammedi W. van Riel C. R. A. (2020). Healthcare service users as resource integrators: investigating factors influencing the co-creation of value at individual, dyadic and systemic levels. J. Serv. Theory Pract. 30, 277–306. doi: 10.1108/JSTP-07-2019-0154

[ref71] Zainuddin N. Previte J. Russell-Bennett R. (2011). A social marketing approach to value creation in a well-women's health service. J. Market. Manag. 27, 361–385. doi: 10.1080/0267257X.2011.547081

[ref72] Zainuddin N. Russell-Bennett R. Previte J. (2013). The value of health and wellbeing: an empirical model of value creation in social marketing. Eur. J. Market. 47, 1504–1524. doi: 10.1108/EJM-10-2011-0564

[ref73] Zainuddin N. Tam L. McCosker A. (2016). Serving yourself: value self-creation in health care service. J. Serv. Market. 30, 586–600. doi: 10.1108/JSM-02-2016-0075

